# Amylose systémique révélée par un goitre amyloïde

**DOI:** 10.11604/pamj.2015.20.278.6597

**Published:** 2015-03-23

**Authors:** Madiha Mahfoudhi, Khaled Khamassi

**Affiliations:** 1 Service de Médecine Interne A, Hôpital Charles Nicolle, Tunis, Tunisie; 2 Service ORL, Hôpital Charles Nicolle, Tunis, Tunisie

**Keywords:** Goitre, amylose systémique, examen immuno-histochimique, goiter, systemic amyloidosis, Immunohistochemical examination

## Image en medicine

L'amylose se caractérise par un dépôt extracellulaire anormal de fibrilles amyloïdes dans les différents tissus. L'apparition d'un goitre secondaire à l'infiltration amyloïde est rare. Il est exceptionnellement révélateur d'une amylose systémique. Son diagnostic est histologique. Patient âgé de 45 ans, aux antécédents de tuberculose pulmonaire traitée et suivi pour une maladie de Crohn, a consulté pour une tuméfaction cervicale antérieure augmentant rapidement de volume et des signes de compression (dyspnée et dysphagie évoluant depuis un mois). L'examen physique a retrouvé un volumineux goitre homogène, ferme et indolore à la palpation. Les aires ganglionnaires étaient libres. L’échographie cervicale a objectivé un goitre homogène. La TDM cervico-thoracique a révélé un goitre homogène arrivant jusqu’à l'orifice supérieur du médiastin avec compression de la filière pharyngo-oesophagienne. Le bilan hormonal thyroïdien était normal. Plusieurs diagnostic ont été évoqués notamment un lymphome ou un carcinome thyroïdien. Le patient a bénéficié d'une thyroïdectomie totale. L'examen anatomopathologique a montré la présence de quelques vésicules thyroïdiennes noyées dans de larges plages éosinophiles amorphes ayant une biréfringence jaune-verdâtre après coloration au rouge Congo en lumière polarisée caractérisant le goitre amyloïde. L'examen immuno-histochimique a conclut à une amylose de type AA. Une recherche systématique d'autres localisations amyloïdes a objectivé une localisation salivaire avec une biopsie de glandes salivaires accessoires positive. Un traitement hormonal substitutif lui a été instauré.

**Figure 1:**
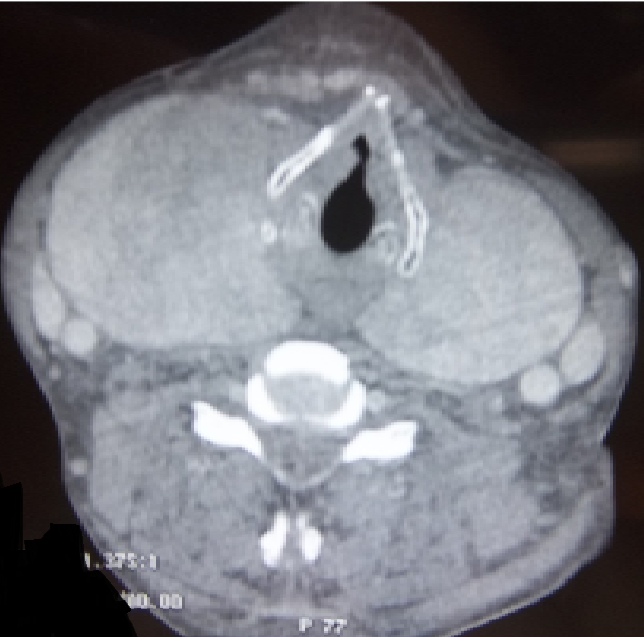
TDM cervico-thoracique en coupe axiale: volumineux goitre homogène arrivant jusqu’à l'orifice supérieur du médiastin

